# Familiarity differentially affects right hemisphere contributions to processing metaphors and literals

**DOI:** 10.3389/fnhum.2015.00044

**Published:** 2015-02-10

**Authors:** Vicky T. Lai, Wessel van Dam, Lisa L. Conant, Jeffrey R. Binder, Rutvik H. Desai

**Affiliations:** ^1^Department of Psychology, University of South CarolinaColumbia, SC, USA; ^2^Department of Neurology, Medical College of WisconsinMilwaukee, WI, USA

**Keywords:** metaphor, right hemisphere, novelty, familiarity, difficulty, laterality, language, imaging

## Abstract

The role of the two hemispheres in processing metaphoric language is controversial. While some studies have reported a special role of the right hemisphere (RH) in processing metaphors, others indicate no difference in laterality relative to literal language. Some studies have found a role of the RH for novel/unfamiliar metaphors, but not conventional/familiar metaphors. It is not clear, however, whether the role of the RH is specific to metaphor novelty, or whether it reflects processing, reinterpretation or reanalysis of novel/unfamiliar language in general. Here we used functional magnetic resonance imaging (fMRI) to examine the effects of familiarity in both metaphoric and non-metaphoric sentences. A left lateralized network containing the middle and inferior frontal gyri, posterior temporal regions in the left hemisphere (LH), and inferior frontal regions in the RH, was engaged across both metaphoric and non-metaphoric sentences; engagement of this network decreased as familiarity decreased. No region was engaged selectively for greater metaphoric unfamiliarity. An analysis of laterality, however, showed that the contribution of the RH relative to that of LH does increase in a metaphor-specific manner as familiarity decreases. These results show that RH regions, taken by themselves, including commonly reported regions such as the right inferior frontal gyrus (IFG), are responsive to increased cognitive demands of processing unfamiliar stimuli, rather than being metaphor-selective. The division of labor between the two hemispheres, however, does shift towards the right for metaphoric processing. The shift results not because the RH contributes more to metaphoric processing. Rather, relative to its contribution for processing literals, the LH contributes less.

## Introduction

Metaphor has been intensely researched for decades, and the view on metaphor has been transformed from it being something poetic reserved for literary use, to something fundamental and generalizable in our daily language and thinking (Lakoff and Johnson, 2003). The pervasiveness of metaphors has been quantified: People use about 5 metaphors for every 100 words of text (Pollio et al., 1990), including 1.8 novel and 4.08 frozen metaphors (e.g., *leg of a table*) per minute of discourse (Pollio et al., 1977). In recent years there has been a surge of interest in studying the neural basis of metaphor, as the answers not only have implications for clinical conditions such as stroke, schizophrenia, and autism, but also have broader impact for understanding the comprehension of language meaning in general.

Perhaps the most debated issue with regard to the neural basis of metaphor is whether the right hemisphere (RH) plays a special role in non-literal language. Several well-known studies reported a special role of the RH in processing metaphors. Winner and Gardner (1977) examined the comprehension of non-literal sentences (e.g., *give me a hand*) in aphasic patients using a sentence-picture matching task. They found that RH patients were less accurate than left hemisphere (LH) patients (accuracies 43% vs. 58%), and suggested that an intact RH is needed for mapping non-literal language meaning onto situations in which it is appropriate (a picture of a person helping others as opposed to a picture of a hand). Bottini et al. (1994) examined the comprehension of new, unusual figures of speech in sentences (e.g., *The investors were squirrels collecting nuts*) in a neurologically healthy sample studied with positron emission tomography. In a semantic judgment task, participants judged whether a given sentence is a plausible metaphor. They compared metaphor and literal conditions and found strongly right-lateralized activation for the metaphor condition in the frontal, temporal, and parietal regions.

However, many functional magnetic resonance imaging (fMRI) studies using neurologically healthy participants have shown that metaphor processing is left lateralized. Rapp et al. (2004) examined novel metaphors in the form of A-is-B (e.g., *Die Worte des Liebhabers sind Harfenklaenge*, “*The lovers’ words are harp sounds*”) and their literal counterparts (*Die Worte des Liebhabers sind Luegen*, “*The lovers’ words are lies*”). In a valence judgment task, participants judged whether a given sentence has a positive or a negative connotation. When compared with a low-level baseline, metaphors led to activation in the right inferior frontal gyrus (IFG) and temporal pole. But when compared with literal sentences, the metaphors only showed activations in the LH, in the left lateral IFG, inferior temporal gyrus, and posterior middle temporal gyrus (MTG). Schmidt and Seger (2009) also examined A-is-B metaphors (e.g., *Respect is a precious gem*). Activations for those metaphors relative to literals were found in the left precentral gyrus, temporal pole, inferior parietal lobe, and lingual gyrus. Chen et al. (2008) examined predicate metaphors embedded in a sentence (e.g., *The man fell under her spell*) in contrast with literals (*The child fell under the slide*). The metaphors led to more activation in the LH than in the RH, with the activations in the left IFG, MTG, and angular gyrus (AG), and the right anterior portion of the MTG.

What, then, determines RH involvement in metaphor processing? One of the most studied factors is metaphor novelty/unfamiliarity.^1^ Electrophysiological studies have shown repeatedly that novel metaphors are processed differently from conventionalized ones (Arzouan et al., 2007; Lai et al., 2009; Lai and Curran, 2013). However, whether this difference is reflected in greater RH involvement is unclear, as electrophysiological metaphoricity effects were very similar between hemispheres (Coulson and Van Petten, 2007). In other studies, novelty has been found to mediate RH activations for metaphors (Mashal et al., 2005, 2007; Stringaris et al., 2006; Schmidt et al., 2007; Pobric et al., 2008). In particular, Faust (2012) proposed that the RH is involved only in novel metaphors, not in conventional metaphors. Mashal et al. (2007) contrasted 2-word conventional (*bright student*) and novel (*pearl tears*) metaphorical expressions with literal (*water drop*) and unrelated (*road shift*) expressions. In a semantic task, participants silently judged if the two words were metaphorically related. Novel metaphors, compared with literals, led to activations in bilateral IFG, right posterior superior temporal gyrus (STG), left middle frontal gyrus (MFG), and middle anterior cingulate gyrus. Conventional metaphors, compared with literals, showed activations in the right postcentral parietal lobe, left posterior STG, and left IFG. Direct comparison between novel and conventional metaphors showed that novelty led to activation in the right posterior superior temporal sulcus (STS), right IFG, and left MFG. Based on these findings, Pobric et al. (2008) conducted a repetitive transcranial magnetic stimulation (rTMS) study to examine the causal role of the right posterior superior temporal region in relation to metaphor processing. They found that rTMS to the right posterior STG impaired the processing of novel metaphors but not conventional metaphors. In contrast, rTMS to the left IFG impaired the processing of conventional but not novel metaphors.

Meta-analyses of imaging studies of non-literal language processing have come to somewhat different conclusions (Bohrn et al., 2012; Rapp et al., 2012; Yang, 2014). In Rapp et al. (2012), the overall metaphors > literal contrast based on 16 studies showed mostly LH activations, in the left parahippocampal gyrus and left IFG, but also some RH activations, such as the right IFG. The conventional metaphors > literal contrast showed activations in the LH only, including the left thalamus, left MTG, left AG, and left IFG. The novel metaphors > literal contrast showed activations in mostly the LH (IFG and MFG) but also in the RH (IFG). In Bohrn et al. (2012), the overall metaphors > literal contrast also led to bilateral activations in the IFG. The conventional metaphors > literal contrast also showed activations in the LH only, including the left IFG, left thalamus, and left STG. The results of the novel metaphors > literal contrast, different from the results of the same contrast in Rapp et al. (2012), showed activations only in the LH, in the left MFG extending into left IFG, and left inferior temporal gyrus. The novel metaphors > literal contrast difference between Rapp et al. (2012) and Bohrn et al. (2012) likely resulted from the inclusion of different studies: Rapp et al. (2012) included 5 studies whereas Bohrn et al. (2012) included 8 studies. Similarly, Yang (2014) observed bilateral activations in IFG for the overall metaphor > literal contrast. In addition, bilateral activations in MFG were also observed for this contrast. The LH activation in the IFG, MFG, inferior parietal lobule (IPL), MTG, and lingual gyrus were observed for the conventional metaphors > literal contrast. As for the novel metaphors > literal contrast, like Rapp et al. (2012) but different from Bohrn et al. (2012), activations were found in RH as well as LH regions, including bilateral IFG, bilateral MFG, left IPL, and right STG.

The present study asks whether it is metaphoricity or novelty that leads to non-specific recruitment of RH areas. Novel or unfamiliar metaphors, and unfamiliar sentences in general, are likely to require more resources involving executive processes related to reanalysis, working memory, inhibition, attention, and decision-making. Unfamiliarity is closely related to the notion of difficulty, which also has been operationalized as reaction times (RTs). If literal sentences are significantly easier to process, they likely do not engage executive processes to the same extent. Consistent with this, several studies reported longer RTs for novel metaphors than their literals: 1385 ms vs. 1261 ms in Mashal et al. (2007), 859 ms vs. 744 ms in the non-TMS group in Pobric et al. (2008), and 2300 ms vs. 2140 ms in Rapp et al. (2004). Novel metaphors also took longer to process than conventional metaphors, e.g., 1385 ms vs. 1275 ms in Mashal et al. (2007) and 859 ms vs. 742 ms in Pobric et al. (2008). Other sentence processing studies have shown that conditions that elicit longer RTs are associated with more activation bilaterally, usually stronger in the LH (e.g., Binder et al., 2005; Desai et al., 2006; Yarkoni et al., 2009; Graves et al., 2010). Thus for items that have longer RTs, it is important to take into consideration the contributions from both hemispheres. If RH contribution is measured only using the activation of the RH, ignoring the potential strong LH activations, then increasing RTs can lead to the (possibly false) conclusion of special contribution of the RH.

Some studies have investigated the role of difficulty in metaphor processing (Monetta et al., 2006; Schmidt and Seger, 2009; Yang et al., 2009; Diaz et al., 2011; Forgács et al., 2012, 2014). Conceptualizing difficulty as task difficulty, Monetta et al. (2006) proposed that metaphors are more difficult to process than literals, which is why the RH is needed for supplying additional resources. They showed that when the task demand is high, neurologically healthy participants comprehended metaphors similarly to patients with RH deficits. Consistent with this proposal, Yang et al. (2009) showed that more difficult conditions led to extensive RH activations including the right IFG, prefrontal cortex, and the temporal and parietal regions. Schmidt and Seger (2009) also examined difficulty, but conceptualized difficulty in terms of the ease of interpretation ratings based on Katz et al. (1988). Comparing difficult metaphors with easy ones, they showed activation in the left IFG. It is unclear whether this activation is due to metaphor-specific processing or general effects of difficulty, because no result on comparable literals (i.e., the difficult literals > easy literals contrast) was reported.

To separate the effects of metaphoric processing from general difficulty effects, unfamiliar metaphors must be compared to similarly unfamiliar literals. A few recent studies included the condition of unfamiliar literals (Diaz et al., 2011; Forgács et al., 2012), but examined metaphors that are of different types compared to those in the current study. Diaz et al. (2011) examined A-is-B type of metaphors (e.g., *A rumor is a disease*) and found that the overall novel > familiar contrast showed activations in the bilateral IFG, parahippocampal gyrus, and posterior MTG. The novel > familiar metaphors surprisingly showed no significant activation, and the novel > familiar literals showed activation in the left IFG. Forgács et al. (2012) examined noun-noun compound metaphors and found that, combining metaphors and literals, the novel > conventional contrast showed activations in regions including left IFG, bilateral insula, and Pre-SMA. The novel > conventional contrasts within the literals and within the metaphors were not reported.

A second issue that is potentially problematic is that metaphors tend to differ from literal sentences in concreteness and imageability. In predicate metaphors, a verb denoting action or motion is often applied to an abstract entity (e.g., *We have to throw out that option*.). Comparable literals require that the action be applied to concrete objects (*We have to throw out that pizza*.) This concreteness confound is difficult to remove, because it reflects inherent differences between metaphors and literals (i.e., applying concrete actions to abstract things is what make it metaphoric). In nominal metaphors, the problem can be the opposite, where metaphors are usually more concrete (*The book was a gem*.) than literals (*The book was excellent*.). Hence, in metaphor-literal comparisons, which brain activations reflect concreteness effects rather than metaphor-specific effects is difficult to determine. A way around this problem is to compare metaphors with other metaphors that differ in their novelty or familiarity. If one assumes that relatively novel metaphors engage metaphor processing machinery to a greater extent, then the novel-familiar contrast can eliminate the concreteness confound. Unfortunately, this introduces another confound, as mentioned above: novel metaphors also use more general cognitive resources. A novel-familiar comparison in literals can be used to differentiate between metaphor-specific and general processes.

In this paper we take this latter approach, and examine the effects of decreasing familiarity of both metaphoric and non-metaphoric sentences. Rather than the dichotomous novel-familiar division, we treat familiarity as a continuous variable, which can potentially provide more power. We use fMRI data from Desai et al. (2011), who tested the role of sensory-motor systems in metaphor comprehension. Their stimuli contained a large set of metaphoric and non-metaphoric sentences that varied in familiarity, including action metaphors (*The council bashed the proposal*), abstract control (*The council criticized the proposal*), and literal action sentences (*The thief bashed the table*). The metaphoric > non-metaphoric contrasts showed activation in the bilateral anterior inferior parietal lobule (aIPL), which has been implicated as an index of (secondary) sensory-motor processing during sentence comprehension. They concluded that the understanding of metaphoric action retains a link to sensory-motor systems involved in action performance. Here we re-analyzed their data with a focus on the issue of laterality.

We also suggest that a potential cause for the divergent findings in the literature lies in the difference in methods of evaluating the role of the RH. In one approach, any activation of the RH (in a metaphor > literal or novel > conventional metaphor comparison) counts as a special role for the RH, regardless of the contribution from the LH (e.g., Schmidt and Seger, 2009). For others, *laterality* of activation is what matters, so that greater RH activation in conjunction with similar or greater LH activation does not count as a special role for the RH (e.g., Coulson and Van Petten, 2007). If the novel > conventional metaphor comparison gives rise to activations in both the RH and LH, then according to the first approach this would be evidence supporting the special role for the RH in metaphor processing. However according to the second approach this would not, unless the novel-conventional difference is greater in the RH than in the LH. Here, we investigate familiarity-related activations in both manners—as activation in the RH and as RH activity in relation to LH activity.

## Materials and methods

We briefly summarize the methods in Desai et al. (2011) and elaborate on the analyses we performed specifically for the current study.

### Participants

Twenty-two right-handed healthy adults (11 women, age 18–33 years, average age 24 years) participated in the imaging experiment. All had normal or corrected-to-normal vision, and none had any neurological disorder. All participants gave informed consent prior to participation. This study was approved by the Institutional Review Board at the Medical College of Wisconsin.

### Materials

Stimuli consist of 81 triplets of sentences, including metaphorical (*The jury grasped the concept*), abstract (*The jury understood the concept*), and literal action sentences (e.g.,* The daughter grasped the flowers*). These sentences were matched in terms of average word frequency; number of phonemes, letters, and syllables; and grammatical structure. In a familiarity norming study, 28 participants rated each sentence on a scale of 1 (not at all familiar) to 7 (very familiar). Items that received lower familiarity ratings were considered more unfamiliar items.^2^ In addition, 81 nonsense sentences, 81 nonword sentences, and 54 sentences with varied syntax were included.

**Figure 1:**
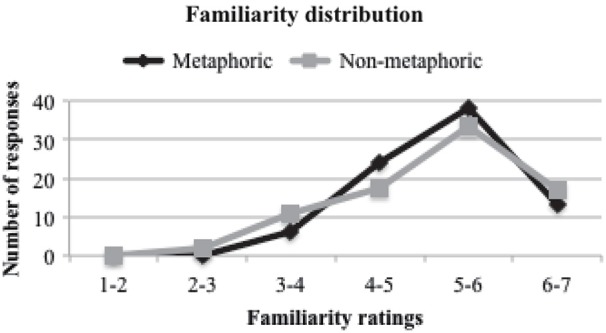
**Distributions of familiarity ratings**.

For the purpose of the present study, the two non-metaphoric sentences (abstract and literal action) were collapsed into a single non-metaphoric condition. The mean familiarity ratings were 5.24 (SD = 0.77) for the metaphoric and 5.17 (SD = 0.98) for the non-metaphoric conditions (*p* = 0.528). Our unfamiliar stimuli were not highly unfamiliar, but were relatively less familiar than the familiar stimuli. The familiarity rating distributions between the metaphoric and non-metaphoric conditions were similar (Figure 1). In a separate meaningfulness judgment task, RTs for each sentence were also collected from 24 subjects. The mean RTs for the metaphoric condition were 1277 ms (SD = 145), which were not statistically different from those for the non-metaphoric condition, 1253 ms (SD = 165; *p* = 0.278). As expected, there was a strong negative correlation between RT and familiarity ratings (*r* = −0.52, *p* < 0.001).

### Experiment procedure and image acquisition

The details of the procedure and image acquisition are described in Desai et al. (2011). Briefly, T2*-weighted whole-brain images were acquired with a TR of 1.8 s and voxel dimensions 3.75 × 3.75 × 4 mm^3^. The sentences were presented visually using white font on a black background, in two parts: The first part was the noun phrase of the sentence (e.g., *The public*), followed by the second part consisting of the verb phrase (*grasped the idea*). The order of sentences was pseudo-randomized. Participants read each sentence and made a covert meaningfulness decision during the imaging experiment. An old/new sentence recognition test was given at the end of each run to encourage and verify subject participation.

### Analysis

AFNI software (Cox, 1996) was used for analyses. In a multiple regression model, we used the mean-centered familiarity rating for each sentence as a condition-specific regressor, to examine areas that are modulated as a function of increasing familiarity. The main effect of familiarity across conditions (metaphoric, non-metaphoric) was computed, showing areas whose response varies with familiarity regardless of metaphoricity. Condition × familiarity interactions were also computed, showing areas that are affected differently by increasing familiarity between metaphoric and non-metaphoric sentences. Given that the right STS has been particularly associated with metaphoric processing (Mashal et al., 2007; Pobric et al., 2008), we also performed a region of interest (ROI) analysis using the right STS, defined based on a maximum probability map created with the Destrieux et al. (2010) parcellation, included with AFNI.

The individual statistical maps and the anatomical scans were projected into standard stereotaxic space (Talairach and Tournoux, 1988) and smoothed with a Gaussian filter of 6 mm FWHM. In a random effects analysis, group maps were created by comparing activations against a constant value of 0. The group maps were thresholded at voxelwise *p* < 0.01 and corrected for multiple comparisons by removing clusters below a size threshold of 1000 mm^3^, to achieve *α* < 0.05. The cluster threshold was determined through Monte Carlo simulations that estimate the chance probability of spatially contiguous voxels exceeding the voxelwise *p* threshold. The analysis was restricted to a mask that excluded areas outside the brain, as well as deep white matter areas and the ventricles.

Additionally, we examined the laterality of activation associated with the main effects and interactions calculated above. A laterality index (LI) was defined as (Q_LH_−Q_RH_)/(abs(Q_LH_)+abs(Q_RH_)), where Q_LH_ and Q_RH_ represent the fMRI-measured LH and RH contributions, respectively, and abs() indicates the absolute value of activation. LI was computed at the whole hemisphere level, and then for ROIs defined by major gyral and sulcal structures defined by a maximum probability map of regions defined by the Desikan-Killiany atlas (Desikan et al., 2006, TT_desai_dk_mpm atlas, provided with AFNI). Rather than choosing a fixed arbitrary threshold to find activated voxels within each ROI, we used the method proposed by Fernández et al. (2001). First, for each participant, the mean of the 5% of the voxels with the strongest absolute value within a (bilateral) ROI were calculated. Active voxels were defined as those that fall within 50% of this mean (on both positive and negative sides) within the ROI. Jansen et al. (2006) found this method to be more robust and reproducible than using voxel counts at a fixed statistical threshold, or using unthresholded activation changes. The total activation of these voxels (defined by the sum of beta-coefficients of all above-threshold voxels) was used to calculate LIs. Both positive and negative correlations were used, as areas correlated positively as well as negatively with familiarity were considered to be relevant to processing of metaphoric or non-metaphoric language.

From the Desikan-Killiany atlas, the middle and inferior frontal gyri, superior and middle temporal gyri (both caudal and rostral divisions), and the posterior STS (“bankssts”) were considered *a priori* regions of interest, as they have been associated with metaphoric processing (Faust, 2012; Rapp et al., 2012). The three divisions of the inferior frontal gyri were combined into a single IFG ROI. Nonparametric Wilcoxon rank-sum tests (Wilcoxon, 1945) were conducted to find LIs that differed from a constant (0), and correction for multiple comparisons was performed using False Discovery Rate (FDR; Genovese et al., 2002).

## Results

### Main effect of familiarity

**Figure 2:**
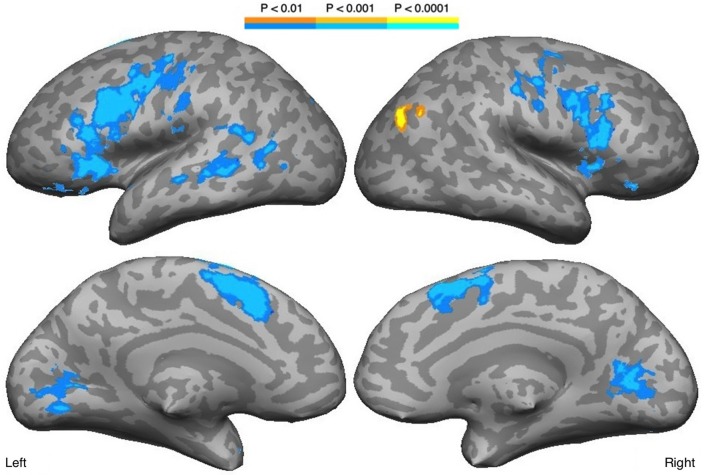
**Regions correlated with familiarity**. Blue scale indicates negative correlation with familiarity, while yellow scale indicates positive correlation.

Decreasing familiarity resulted in increased activation in both hemispheres with LH dominance, but with some activation in the RH (Figure 2, Table 1). These regions included bilateral IFG, IFS, MFG, insula, precentral gyrus and central sulcus, lateral orbital gyrus, medial SFG, lingual gyrus, and cuneus. The STS and MTG were activated in the LH. A positive correlation with familiarity was observed in the right posterior AG. The ROI analysis on the right STS did not reveal any activation.

**Table 1:** **Regions showing a main effect of decreased familiarity. Cluster volume (in mm^3^), maximum z-score, and the coordinates in Talaraich space are shown**.

Volume	Max	*x*	*y*	*z*	Structure
24960	−5.6	−42	−4	36	L inf frontal g and s, precentral g, mid frontal g
	−4.4	−29	24	13	L inf frontal g, insula
	−4.0	−38	−12	61	L precentral g, mid frontal g
	−3.7	−47	−26	26	L supramarginal g
	−3.5	−29	22	−18	L orbital g, temporal pole, sup temporal g
11950	−4.7	52	16	22	R inf frontal g, mid frontal g, precentral g
	−3.8	49	5	47	R mid frontal g, precentral g
	−3.8	27	−11	46	R precentral g
	−3.8	53	−16	40	R postcentral g, precentral g, supramarginal g
	−3.8	29	20	12	R insula, inf frontal g
	−3.8	45	24	−8	R orbital g, sup temporal g, inf front pars orbitalis
9811	−5.1	4	13	50	R inf frontal g, sup temporal g, temporal pole
5761	−4.1	18	−66	4	R lingual g, cuneus
	−3.8	−16	−72	1	L lingual g, cuneus
	−3.0	−23	−72	25	L intraparietal s, precuneus, cuneus
4100	−3.8	−47	−49	14	L sup temporal s, mid temporal g, inf pariet lobule
	−3.5	−45	−19	−2	L sup temporal g and s, insula, mid temporal g
2046	4.7	36	−83	34	R mid occipital g, angular g, sup occipital g
1252	−3.7	18	−74	−25	R cerebellum

*L = left hemisphere, R = right hemisphere, g = gyrus, s = sulcus, sup = superior, mid = middle, inf = inferior*.

### Interaction with metaphoricity

No regions showed familiarity × metaphoricity interaction in the whole brain analysis, nor was there any familiarity × metaphoricity interaction in the right STS ROI.

### Laterality analysis results

Laterality analysis of the main effect of familiarity based on predefined ROIs (as opposed to the voxelwise activations found above) showed that MTG becomes more left lateralized as familiarity is decreased across both sentence types (Table 2). The posterior STS and caudal MFG also showed marginal left lateralization. No region showed right lateralization.

**Table 2:** **Laterality indices for regions showing main effect of correlation with familiarity (positive values = left lateralization; negative values = right lateralization)**.

Structure	Median LI metaphoric	Median LI non- metaphoric	Wilcoxon V	Main effect *p* (two-tail, corrected)
Mid temporal g	0.12	0.34	174.0	0.042*
Post sup temporal s	0.21	0.31	185.0	0.058
Caudal mid frontal g	0.03	0.38	164.5	0.088

* indicates p < 0.05. Regions showing a trend (p < 0.1) are also shown.

**Figure 3:**
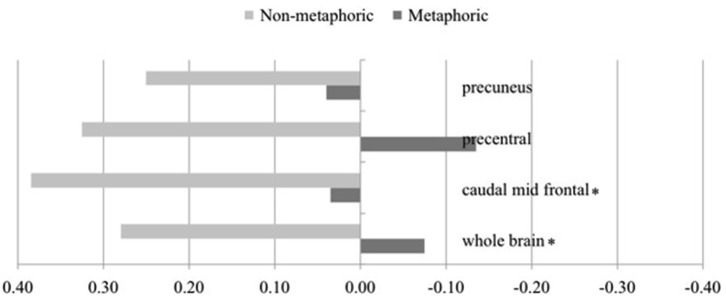
**Laterality indices for regions showing metaphoricity × familiarity interaction, depicted for metaphoric and non-metaphoric conditions (positive values = left lateralization; negative values = right lateralization)**. * indicates *p* < 0.05. Regions showing a trend (*p* < 0.1) are also shown.

The critical analysis involves the familiarity × metaphoricity interaction using the LIs for both conditions. Because the hypothesis predicts greater right laterality for metaphors, we examined this effect with one-tailed tests to gain more sensitivity. This analysis showed that the more unfamiliar a metaphoric item is, the more right lateralized it becomes relative to increasingly unfamiliar non-metaphoric sentences, at the whole brain level and also in the caudal MFG (Table 3). The interaction in both regions arose from a strong left lateralized activation for the non-metaphoric sentences and no lateralization (non-significantly different from 0) for the metaphoric sentences (Figure 3). Interaction trends were observed in the precuneus and precentral gyrus, following the same pattern (left lateralization for non-metaphors, no lateralization for metaphors).

**Table 3:** **Laterality indices for regions showing metaphoricity × familiarity interaction (positive values = left lateralization; negative values = right lateralization)**.

Structure	Median LI metaphoric	Median LI non-metaphoric	Wilcoxon V	Interaction *p* (one-tail, corrected)
Whole brain	−0.08	0.27	56.0	0.011*
Caudal mid frontal g	0.03	0.38	47.0	0.047*
Precentral g	−0.14	0.33	47.0	0.062
Precuneus	0.04	0.25	50.0	0.062

Regions showing a trend (p < 0.1) are also shown. * indicates p < 0.05.

## Discussion

We examined the effects of decreasing familiarity on both metaphoric and non-metaphoric sentences, to examine the extent to which RH activations for relatively novel, unfamiliar metaphors are driven by the general cognitive demands for processing unfamiliar stimuli. We found first that decreased familiarity led to increased activation in both the left and RHs regardless of metaphoricity, with greater activation in the LH. This is consistent with the greater LH activation found in some studies that argue against a special role for RH (e.g., Rapp et al., 2004, 2012; Bohrn et al., 2012). While the controversy relates only to the RH, the LH can also be argued to play a “special role” in processing unfamiliar metaphors and literals, likely reflecting a greater use of the existing left lateralized language system.

While overall the unfamiliarity-related activations were left lateralized, some RH regions were also found to respond to decreased familiarity across both sentence types, most notably the right IFG, MFG, and insula. This pattern suggests that activation in these regions, frequently reported in metaphor studies and used as evidence for a special role of the RH in metaphor processing, is unlikely to reflect metaphor-specific processing but instead reflects increased general cognitive demands of processing unfamiliar stimuli. Past studies have implicated the IFG for processing difficulty (Yang et al., 2009), though in contrast to the right IFG activation observed in the present study, increased difficulty has been associated both with left (Schmidt and Seger, 2009) and bilateral (Diaz et al., 2011) IFG activation. The MFG was activated to a greater extent in the easier condition in Schmidt and Seger (2009) and was left lateralized. These differences might have resulted from a difference in the degree of unfamiliarity of the tested items in these studies: Our items were congregated closer to the familiar end of the scale whereas the items in Schmidt and Seger (2009) were closer to the unfamiliar end.

Our finding that a left lateralized network is engaged for unfamiliar sentences meshes well with Cardillo et al. (2012). In this study, the authors manipulated familiarity parametrically by exposing participants to novel metaphoric stimuli to different degrees. Effects of decreasing familiarity were found in the bilateral IFG, left posterior MTG, and right postero-lateral occipital gyri. This study did not include the corresponding literal conditions of varying familiarity. Nonetheless, the fact that both familiarity induced within a session (Cardillo et al., 2012) and familiarity established through lifelong experiences (the current study) found LH activation further support the view in which the LH and in particular the left IFG are involved in processing unfamiliar stimuli due to general cognitive demands.

We also examined RH contributions relative to LH contributions, by computing laterality indices. Greater left lateralization was observed in the MTG and marginally in the posterior STS. These regions are commonly associated with language processing, including semantic, combinatorial/syntactic, and phonological processing (Binder et al., 1997, 2009; Friederici, 2002; Hartwigsen et al., 2010; Price, 2012). These results are consistent with greater involvement of left-dominant language systems for dealing with more difficult or unfamiliar sentences.

Turning to the laterality analysis of the metaphor × familiarity interaction, the laterality of the unfamiliarity-related activation at the whole brain level shifted to the right for metaphors relative to non-metaphors. This interaction arose from left lateralization of non-metaphors, and no lateralization (both hemispheres being activated statistically equally, with small numerical right lateralization) for metaphors. Thus, while the RH itself is not activated more than the LH for unfamiliar metaphors relative to familiar metaphors, its contribution is relatively greater for unfamiliar metaphors than for unfamiliar non-metaphors. These results suggest that metaphoric processing alters the division of labor between the hemispheres, with more bilateral activation as opposed to left lateralized activation for non-metaphors. In other words, the RH does not contribute to a greater extent in metaphoric processing, but the LH contributes less.

The caudal MFG also showed this interaction, arising from the same pattern of left lateralization for non-metaphors and bilateral activity for metaphors. Middle frontal gyri have been associated with working memory (Leung et al., 2002), inhibitory control (Garavan et al., 1999), sustained attention and verification (Kanwisher and Wojciulik, 2000; Cabeza et al., 2003; Habib et al., 2003). While these processes are by no means metaphor specific, they appear to be engaged more for processing unfamiliar metaphoric sentences than for unfamiliar non-metaphoric sentences. The precentral gyrus and precuneus showed a marginal interaction, with more bilateral processing for metaphors. The precentral gyrus activation may be related to the semantic content of the action metaphors used in the current study. The precuneus has been implicated in mental imagery strategies and episodic memory retrieval (Cavanna and Trimble, 2006), which are relevant for metaphor processing.

A few theories predict the RH involvement in processing unfamiliar or novel stimuli. One prevailing view of the RH is that it maintains a wider semantic field, and keeps alternative meanings and senses active (Beeman and Chiarello, 1998). The putative special role of the RH in metaphor processing involves enabling access to these alternative senses. Another claims that while the processing of formulaic language like idioms are primarily left lateralized, the RH can control or modulate this processing (Van Lancker Sidtis, 2012, p.352). And yet another view suggests that the RH is involved in non-salient meaning processing (Giora, 2003). We suggest an additional possibility, namely that the RH, and especially regions such as the right IFG, come online when the resources provided by the LH are not sufficient due to difficulty of comprehension. For all types of difficult linguistic stimuli, the LH is activated more, and there is a “spill over” effect in the RH. This may also explain why in older individuals, more bilateral activity is often observed. With diminished efficiency and capacity of the aged brain, “assistance” from the RH is needed. The bilateral nature of increased activation here, and in several studies that reported regions correlated with RT cited earlier, also supports this idea. We are not aware of any studies that show increased RH activation for language processing without also showing increased LH activation.

While we have focused on the effects of decreased familiarity, increased familiarity showed more activation in the right AG. The right AG is part of the semantic system, showing greater activation for more meaningful relative to less meaningful linguistic stimuli. (Binder et al., 2009). Graves et al. (2010) found activation in the same region for meaningful word combinations (*flower girl*) relative to word pairs that are difficult to combine into a whole (*girl flower*) in a semantic judgment task. An interpretation consistent with these observations is that the activation in the right AG reflects the semantic richness of familiar word combinations, and spreading activations due to greater associations with more meaningful complex stimuli.

One characteristic of the current study is that the stimulus set did not include highly novel metaphors, and mostly included somewhat familiar, comprehensible metaphors of the kind that would be expected in daily language and popular media. This also means that the “unfamiliar” sentences in the current study may be better treated as “less familiar” sentences. It is possible that RH involvement changes for highly unfamiliar metaphors requiring extensive analysis, but this can only be assessed in comparison to equally odd, unfamiliar, or difficult non-metaphoric language. The range of unfamiliarity explored in this study may also be more relevant and ecologically valid, as majority of metaphors encountered in daily or routine language processing are likely created to be comprehensible without extensive analysis. We speculate that very novel or odd metaphors are not only rare, but may necessitate qualitatively different mechanisms involving conscious cognitive control that are usually not engaged during most language processing.

Another characteristic of the study is that the metaphors were embedded in sentences, and familiarity ratings were obtained for whole sentences (*The public grasped the idea*). The sentences were not arbitrarily complex, but had a fixed structure involving a noun phrase (an article and a noun) preceding the metaphor. The effects observed here may also represent some contributions from the noun phrase (*The public*), although those were also present in the non-metaphoric sentences (*The public understood the idea*). The studies that use two-word combinations or A-is-B metaphors have an advantage that the entire stimulus constitutes the metaphor. On the flip side, most metaphors are also encountered in sentence (and larger) contexts, and not in isolation, in routine language processing. The larger context, and the noun phrase in this case, can affect how readily a given metaphor is comprehended (e.g., the metaphor in *The student grasped the idea* may behave like a slightly more familiar metaphor than the metaphor in *The cook grasped the idea*, because students have a stronger association with understanding things). Thus results pertaining to how metaphors are processed and modulated in sentence contexts (and in the minimal noun phrase context in this case) are also relevant to metaphor processing.

## Conclusions

With decreased familiarity or increased novelty, there is greater activation in the whole brain across both metaphors and non-metaphors, with more extensive recruitment in the LH. Some regions in the RH, especially the IFG and insula, respond to decreased familiarity. Activation of the right IFG, a consistent finding in studies of metaphors, likely reflects a general difficulty effect and not metaphor-specific processing. These findings suggest it is important to equate the novelty/unfamiliarity of the stimuli in studies of metaphor processing. Comparisons of novel and conventional metaphors, or novel metaphors and conventional literal sentences can, and usually do, lead to confounds due to greater general cognitive demands of processing unfamiliar stimuli.

In the present study, no brain regions responded selectively to the decreasing familiarity of metaphors. Unfamiliarity-related recruitment of the right and LHs is relatively bilateral for metaphors and left lateralized for non-metaphors, suggesting a *relatively* greater role for the RH in processing unfamiliar metaphors compared to non-metaphors. Thus, the RH does not contribute to a greater extent in metaphoric processing in an absolute sense, but LH contributes less, affecting lateralization. The answer to the question “does the RH play a special role in metaphor processing?” is both “yes” and “no”. It is “yes” in the sense that relative to the LH, the RH does show greater activation compared to its relative activation for processing non-metaphoric stimuli. It is “no” in the sense that the magnitude of activation in the RH, taken by itself, is similar for both metaphoric and similarly-difficult non-metaphoric stimuli.

## Conflict of interest statement

The authors declare that the research was conducted in the absence of any commercial or financial relationships that could be construed as a potential conflict of interest.

## Footnotes

^1^Some studies use the term “novel” (e.g., Faust, 2012) whereas others use “unfamiliar” (e.g., Schmidt et al., 2007). In this paper we used the two interchangeably. When we review the work of others, we use the terminology that they chose to use, which potentially represent these authors’ theoretical stance. We treat “novel” not in a categorical sense (a metaphor that could not have been encountered before), but in a more continuous sense, as equivalent of “unfamiliar”. That is, we treat novelty and familiarity as ends of the same continuous scale.

^2^All sentences are available at http://www.mccauslandcenter.sc.edu/delab/?attachment_id=302
